# Abdominal injuries in a major Scandinavian trauma center – performance assessment over an 8 year period

**DOI:** 10.1186/1752-2897-8-9

**Published:** 2014-08-02

**Authors:** Sigrid Groven, Christine Gaarder, Torsten Eken, Nils Oddvar Skaga, Paal Aksel Naess

**Affiliations:** 1 Department of Traumatology, Division of Emergencies and Critical Care, Oslo University Hospital Ulleval, Nydalen, PO Box 4956, Oslo N-0424, Norway; 2 Department of Surgery, Vestre Viken HF Drammen Hospital, Oslo, Norway; 3 Department of Anaesthesiology, Division of Emergencies and Critical Care, Oslo University Hospital, Ulleval, Oslo, Norway

**Keywords:** Trauma, Abdominal injury, Laparotomy, Non-operative management, Performance improvement, Variable life-adjusted display, Survival

## Abstract

**Introduction:**

Damage control surgery and damage control resuscitation have reduced mortality in patients with severe abdominal injuries. The shift towards non-operative management in haemodynamically stable patients suffering blunt abdominal trauma has further contributed to the improved results. However, in many countries, low volume of trauma cases and limited exposure to trauma laparotomies constitute a threat to trauma competence. The aim of this study was to evaluate the institutional patient volume and performance for patients with abdominal injuries over an eight-year period.

**Methods:**

Data from 955 consecutive trauma patients admitted in Oslo University Hospital Ulleval with abdominal injuries during the eight-year period 2002-2009 were retrospectively explored. A separate analysis was performed on all trauma patients undergoing laparotomy during the same period, whether abdominal injuries were identified or not. Variable life-adjusted display (VLAD) was used in order to describe risk-adjusted survival trends throughout the period and the patients admitted before (Period 1) and after (Period 2) the institution of a formal Trauma Service (2005) were compared.

**Results:**

There was a steady increase in admitted patients with abdominal injuries, while the number of patients undergoing laparotomy was constant exposing the surgical trauma team leaders to an average of 8 trauma laparotomies per year. No increase in missed injuries or failures of non-operative management was detected. Unadjusted mortality rates decreased from period 1 to period 2 for all patients with abdominal injuries as well as for the patients undergoing laparotomy. However, this apparent decrease was not confirmed as significant in TRISS-based analysis of risk-adjusted mortality. VLAD demonstrated a steady performance throughout the study period.

**Conclusion:**

Even in a high volume trauma center the exposure to abdominal injuries and trauma laparotomies is limited. Due to increasing NOM, an increasing number of patients with abdominal injuries was not accompanied by an increase in number of laparotomies. However, we have demonstrated a stable performance throughout the study period as visualized by VLAD without an increase in missed injuries or failures of NOM.

## Introduction

Damage control surgery and damage control resuscitation have reduced mortality in patients with severe abdominal injuries [1,2]. The shift towards non-operative management (NOM) in haemodynamically stable patients suffering blunt abdominal trauma has further contributed to the improved results [3-6]. However, in many countries low volumes of trauma cases and limited exposure to trauma laparotomies combined with increasing subspecialization constitute a threat to education and competence with this patient group [7-16].

The above description seems applicable to Norway as well, where Oslo University Hospital Ulleval (OUH-U) is the only equivalent to a Level I trauma centre. In 2000-2002, based on the existing trauma centre infrastructure, protocols were revised and educational programs improved. Moreover, new treatment protocols for abdominal compartment syndrome, temporary abdominal closure and solid abdominal injuries including angiographic embolization were introduced, the latter leading to increased NOM rates [17,18]. During the same period, our surgical trauma team leaders participated in no more than 10 trauma laparotomies annually despite a high percentage of severely injured patients admitted [19].

This study was undertaken to assess the institutional patient volume and performance over the period 2002-2009 for patients with abdominal injuries including the use of variable life-adjusted display (VLAD) in order to describe risk-adjusted survival trends throughout the period.

## Patients and methods

OUH-U is a major Scandinavian trauma centre currently admitting approximately 1 800 trauma patients per year. It serves as a regional trauma centre for 2.7 million people, more than half the Norwegian population. Blunt trauma is the mechanism of injury in 90% of the patients. Consistently, approximately 40% [20] are severely injured with an injury severity score (ISS) >15 [21].

The current study is a retrospective analysis of all patients in the OUH-U trauma registry admitted from January 1, 2002 to December 31, 2009 with abdominal or diaphragmatic injury grade ≥ 2 according to the Abbreviated Injury Scale 1990 Revision, update 98 (AIS 98) [22]. In order to assess surgeons’ exposure to operative trauma care, a separate analysis was performed on a population consisting of all trauma patients undergoing laparotomy during the same period, whether abdominal injury was identified or not. We have previously described the surgical trauma team leader role in our institution as filled by experienced general surgeons at the end of their surgical subspecialization, but most often with limited trauma experience [19]. The trauma team leaders attend an extensive training program, but typically stay in the role as trauma team leader for only 1.5 years due to the time limits of their training appointment.

Based on a recently published study from our institution in which we demonstrated increased survival from 2005 for the total trauma population [20], we chose to compare the period before (period 1; 2002 to 2004) and after that time point (period 2, 2005 to 2009).

Patients were identified from the OUH-U Trauma Registry and data extracted included age, gender, mechanism of injury, ISS, Glasgow coma scale (GCS) score, surgical procedure codes, probability of survival (Ps) calculated using TRISS methodology [21] with National Trauma Data Bank 2005 (NTDB 05) coefficients, hospital length of stay (LOS), 30-day survival [23], and main cause of death. The OUH-U Trauma Registry, which has been operational since August 2000, includes all trauma patients admitted through trauma team activation (irrespective of ISS), or with penetrating injuries proximal to elbow or knee, or with head injury AIS ≥ 3, or with ISS ≥ 10 admitted to OUH-U directly or via a local hospital within 24 hours after injury. Patients with an isolated single extremity fracture and transfers more than 24 hours after injury are included only if the trauma team is activated. In cases where patients were intubated and anaesthetized before admission, GCS score and respiratory rate were recorded as the values documented immediately prior to intubation. For the population undergoing laparotomy, patient charts were used to extract data on surgical procedures, failure of NOM, missed injuries and non-therapeutic laparotomies. Patients undergoing laparotomy before transfer to OUH-U were excluded from this analysis. Failure of NOM was defined as any laparotomy in patients where the intention after initial work-up had been that of NOM. Missed injury was defined as an injury not recognized at the completion of the initial work-up and treatment, but later leading to a therapeutic procedure. A laparotomy was deemed non-therapeutic by the absence of intra-abdominal injury necessitating surgical intervention. In cases of doubt regarding categorization of laparotomies, the three authors with surgical competence reached consensus.

### Data analysis

Period 1 and period 2 were compared for demographics and 30-day mortality, first for the total population with diagnosed abdominal injuries with AIS ≥ 2, and subsequently for the population of patients undergoing laparotomy.

VLAD was used in order to describe risk-adjusted survival trends throughout the eight-year period. VLAD is a refinement of the cumulative sum method that adjusts death and survival by each patient’s risk status (probability of survival, Ps), providing a graphical display of performance over time [24]. Every patient was assigned a value corresponding to gained or lost fractional life. Each survivor contributed a reward of 1 – Ps and each death a penalty of – Ps. Starting from zero, each patient’s contribution in terms of reward or penalty was added to the summed contribution of all previous patients and the resulting number plotted vs. time of patient admission. This plot of cumulative sum of penalties and rewards shows the difference between expected and actual cumulative mortality over time, i.e., the number of excess saved lives compared to the reference model (TRISS with NTDB 2005 coefficients) since the first patient was admitted. A linear VLAD graph thus indicates stable performance while an upward deflection suggests improved standards of care and a downward deflection indicates a decline in performance. Consequently, the relation to the chosen reference model is less interesting than changes in trend over time in the studied population [24].

W-statistics, expressing excess survivors per 100 patients treated at OUH-U compared to TRISS model prediction, were calculated according to convention and used to compare outcomes for the two periods [25]. Non-overlapping 95% confidence intervals were deemed as significant differences between groups.

Fisher’s Exact test was used for analyses of categorical data, and Student’s *t* test and Mann-Whitney *U* test were used for normally and non-normally distributed continuous data, respectively.

Statistical analyses were performed using PASW Statistics 18 statistical software (SPSS Inc., Chicago, USA) and StatView 6.5 statistical software (SAS Institute, Inc., Cary, USA). A *p* value of < 0.05 was considered to indicate significance.

The study was approved by the Institutional Data Protection Officer, and the Regional Committee for Medical Research Ethics had no objections.

## Results

**Figure 1:**
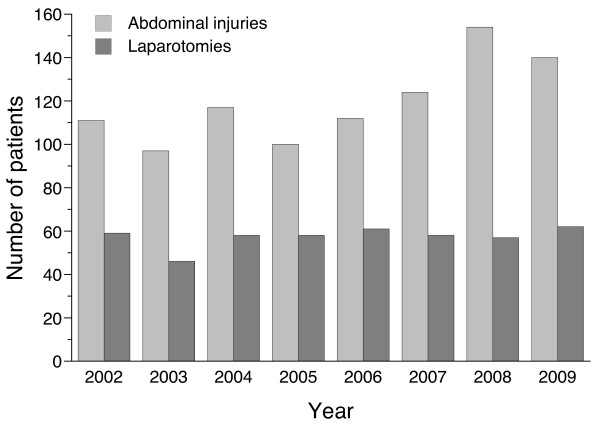
Year by year numbers of patients with abdominal injury and patients undergoing laparotomy.

A total of 955 patients with abdominal injury AIS grade ≥ 2 were identified, 325 patients in period 1 and 630 patients in period 2. Demographics are shown in Table 1. A total of 459 trauma patients were identified to have undergone laparotomy, 163 patients in period 1 and 296 patients in period 2 (Table 2). The high ISS and mortality rates reflect that many of these patients were polytraumatized with severe extraabdominal injuries.In spite of an increase in admitted patients with abdominal injuries in period 2, the annual number of patients undergoing laparotomy was remarkably stable throughout the study period (Figure 1). With one night in seven on call the trauma team leaders participated in an average of 8 trauma related laparotomies per year.

**Table 1:** Comparison of Period 1 and 2 for patients with abdominal injury

	**Period 1**	**Period 2**	***p***
	**n = 325**	**n = 630**	
Age, mean	34.6	33.4	0.36
Male (%)	243 (75)	453 (72)	0.36
Blunt (%)	281 (86)	542 (86)	0.92
GCS score, median (quartiles)	15.0 (7.5-15.0)	15.0 (11.8-15.0)	<0.01
ISS, median (quartiles)	29.0 (17.0-42.0)	26.0 (16.0-38.0)	0.05
LOS, median (quartiles)	6.0 (3.0-11.0)	8.0 (4.0-14.0)	<0.01
Deaths (%)	63 (20)	65 (10)	<0.01
W NTDB 05 (95% CI)	4.14 (1.93 to 6.36)	6.77 (4.83 to 8.71)	

GCS: Glascow Coma Scale; ISS: injury severity score; LOS: length of stay; W NTDB 05: W-statistics with coefficients from National Trauma Data Bank 2005; CI: confidence interval.

One patient from period 2 has missing data on age, mechanism of injury, GCS and ISS.

**Table 2:** Comparison of Period 1 and 2 for patients undergoing laparotomy

	**Period 1**	**Period 2**	***p***
	**n = 163**	**n = 296**	
Age, mean	37.4	36.6	0.63
Male (%)	127 (78)	220 (74)	0.42
Blunt (%)	99 (61)	183 (62)	0.84
GCS score, median (quartiles)	15.0 (5.0-15.0)	15.0 (9.0-15.0)	0.09
ISS, median (quartiles)	29.0 (10.0-45.0)	25.0 (10.0-38.0)	0.02
LOS, median (quartiles)	5.0 (2.0-11.0)	8.0 (3.0-16.0)	<0.01
Deaths (%)	60 (37)	59 (20)	<0.01
W NTDB 05 (95% CI)	0.25 (-3.81 to 4.30)	3.91 (0.99 to 6.84)	

GCS: Glascow Coma Scale; ISS: injury severity score; LOS: length of stay; W NTDB 05: W-statistics with coefficients from National Trauma Data Bank 2005; CI: confidence interval.

One patient from period 2 has missing data on age, mechanism of injury, GCS and ISS.

**Figure 2:**
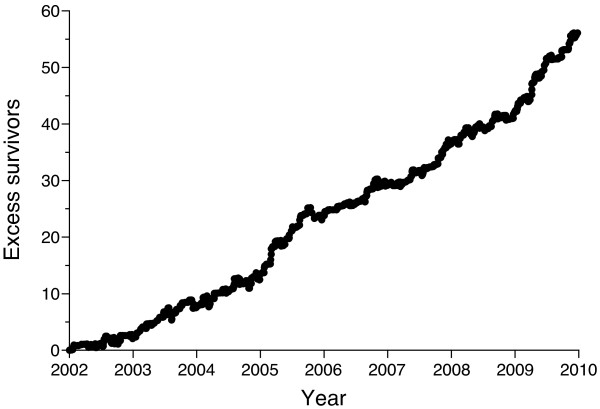
Cumulative excess survival for patients with abdominal injury.

Unadjusted mortality rates decreased from period 1 to period 2 for all patients with abdominal injuries and for the population undergoing laparotomy, and was accompanied by an increase in LOS in both groups (Tables 1 and 2). However, an increase in GCS score and a decrease in ISS could be detected between the periods, indicative of a less injured patient population in period 2. The significant increase in survivors from period 1 to period 2 was not accompanied by a corresponding reduction in risk-adjusted mortality (W-statistics), as demonstrated by overlapping confidence intervals (Tables 1 and 2). In accordance with this, VLAD demonstrated a steady performance throughout the study period (Figure 2).

Main causes of death for patients undergoing laparotomy are listed in Table 3, showing bleeding as the main cause of death in both periods, followed by head injury and sepsis/MOF.

**Table 3:** Main causes of death for patients undergoing laparotomy

	**Period 1**		**Period 2**		
	**N**	**%**	**N**	**%**	p*
Bleeding	36	60	37	63	0.85
Sepsis/MOF	7	12	6	10	1.00
Head injury	11	18	8	13.5	0.62
Other/unknown	6	10	8	13.5	0.58
Total	60	100	59	100	

MOF: multiple organ failure.

*Fisher’s Exact tests for each individual cause against the sum of all other causes.

There was a significant reduction in failure of NOM and a trend toward decreasing non-therapeutic laparotomies and missed injuries in period 2 (Table 4). Approximately half of the patients that underwent non-therapeutic laparotomies in both periods (26/51 in period 1 and 38/80 in period 2; p = 0.72) had sustained penetrating trauma.

**Table 4:** Missed injuries, NOM failures and non-therapeutic laparotomies

	**Period 1**	**Period 2**	***p***
	**n = 163**	**n = 296**	
Missed injuries (%)	9 (6)	13 (4)	0.65
Failure of NOM (%)	9 (6)	5 (2)	0.04
Non-therapeutic laparotomies (%)	51 (31)	80 (27)	0.33

NOM: non-operative management.

## Discussion

The present report demonstrates a limited exposure to abdominal injuries and trauma laparotomies in our hospital. One could fear that such a low exposure, now largely depicted as part of the “future of trauma”, might jeopardize education and clinical competence and result in deteriorating performance [8,11,16,26-28]. However, a stable performance over the study period for patients with abdominal injuries was demonstrated by VLAD. The increase in patient volume was not accompanied by an increase in the number of laparotomies. However, there was no increase in missed injuries or failures of NOM.

In 2000-2002, based on the existing trauma centre infrastructure, protocols were revised and educational programs improved. Moreover, new treatment protocols for abdominal compartment syndrome, temporary abdominal closure and solid abdominal injuries including angiographic embolization were introduced, the latter leading to increased NOM rates [17,18]. In a recently published study using VLAD on all trauma patients entered in the institutional trauma registry during the period 2002-2008 we demonstrated that the start of a long-lasting performance improvement with increased survival coincided with the formalization of a dedicated trauma service in 2005 [20]. The absence of a similar distinct change in performance for the subpopulation with abdominal injuries is likely caused by the above mentioned fact that most changes in protocols and education affecting the treatment of abdominal injuries specifically occurred before 2002. The reduction in crude mortality was accompanied by an increase in LOS. In addition to fewer deaths causing very short LOS, this change is most likely caused by a 5 bed increase in surgical ICU capacity over the study period. Patients previously transferred to their local hospital at an earlier stage could be kept in the trauma center when deemed beneficial in order to optimize care. NOM for blunt trauma might lead to delayed diagnosis and treatment of hollow viscus injury, and such delays have been associated with increased mortality [29-32]. In spite of the high NOM rate in our material, the rate of missed injuries remained low throughout the study period.

Our protocol mandated immediate laparotomy in haemodynamically compromised patients with verified or suspected ongoing abdominal bleeding or patients with peritonitis. Patients with suspected or verified hollow organ injury or diaphragmatic disruption based on radiological findings underwent laparotomy. A significant number of laparotomies performed in both periods were non-therapeutic (Table 4). This may be explained by the finding in both periods that half of these patients had sustained penetrating trauma. It was only in 2007 that a protocol allowing observation of haemodynamically stable patients with abdominal stab wounds was implemented. Thus, an effect on the non-therapeutic laparotomy rate is not expected to be visible in the current study.

Some weaknesses should be commented on in addition to those associated with the retrospective nature of the study. The study addresses two consecutive periods, with the possibility of other factors influencing outcome measures as part of the ongoing quality improvement program, such as the implementation of the updated massive transfusion protocol in 2007, as well as a revised protocol for the treatment of severe traumatic brain injury in 2009. Differences in case mix and changes in patient volume are factors that could influence outcome independent of institutional performance. However, such differences are adjusted for in the survival prediction model. The categorization of laparotomies as being therapeutic or not, injuries as being missed, and failures of NOM are subject to some degree of subjectivity. However, the same commonly used definitions were applied in both periods, and in cases of doubt, the three authors with surgical competence reached consensus.

A VLAD chart has some weaknesses calling for cautious interpretation. The method is dependent on the rate of data collection, e.g., the slope of the curve representing excess survivors over time will increase if more patients are included per time unit even when performance is unchanged, provided that it is better than the underlying survival prediction model. However, when plotted against patient numbers instead of time, the visual impression remains the same for our patient population. Furthermore, any short-term change can be due to random error. Additionally, as for any survival analysis, deficiencies in trauma care not leading to death will escape detection. However, the obvious benefit of VLAD is its ability to detect both positive and negative changes in clinical performance at an early stage and we recommend VLAD as a valuable instrument for trauma auditing purposes that should be more widely used.

Even in a high volume trauma center the exposure to abdominal injuries and trauma laparotomies is limited. Due to increasing NOM, an increasing number of patients with abdominal injuries was not accompanied by an increase in number of laparotomies. However, we have demonstrated a stable performance throughout the study period as visualized by VLAD without an increase in missed injuries or failures of NOM.

## Competing interest

The authors declare that they have no competing interests.

## Authors’ contributions

SG: Study design, collected and extracted data, performed statistical analysis, generated illustrations, drafted the manuscript and interpreted the data, co-wrote and critically reviewed the manuscript. CG: Study design, drafted the manuscript and interpreted the data, co-wrote and critically reviewed the manuscript. TE: Performed the VLAD analyses, revised all statistics, co-wrote and critically reviewed the manuscript. NOS: Extracted data from the OUH Trauma Registry, performed the W-statistics analyses, co-wrote and critically reviewed the manuscript. PAN: Study design, drafted the manuscript and interpreted the data, co-wrote and critically reviewed the manuscript. All authors have read and approved the final manuscript.
